# Beyond traditional staging system: Tumor-Node-Metastasis-Blood staging system improves treatment decisions and prognostic stratification in cancer patients

**DOI:** 10.3389/fonc.2026.1742160

**Published:** 2026-03-27

**Authors:** Yue Li, Yuanyuan Zhan, Cheng Shen

**Affiliations:** 1 West China Clinical Medical College of Sichuan University, Chengdu, China; 2 Department of Thoracic Surgery, West China Hospital, Sichuan University, Chengdu, China

**Keywords:** cutaneous T-cell lymphoma, lung cancer, molecular biomarkers, precision oncology, TNMB staging system

## Abstract

**Background:**

The traditional Tumor-Node-Metastasis (TNM) staging system for solid tumors relies on anatomical assessment but possesses inherent limitations in capturing systemic micrometastases and molecular-level disease burden. Recently, the Tumor-Node-Metastasis-Blood (TNMB) staging system has garnered significant attention. It aims to provide a systematic synthesis of the origin of the TNMB staging system, its current applications in tumors, and its future prospects, while evaluating its potential value in enhancing early diagnosis, prognostic assessment, and the precision of therapeutic decision-making.

**Methods:**

A systematic literature search was conducted in PubMed and Web of Science databases from 2018 to 2026 using keywords related to neoplasms and TNMB staging system. Articles were screened for inclusion, and 21 studies focusing on cutaneous T-cell lymphoma and lung cancer were included for analysis.

**Results:**

The TNMB staging system enhances risk stratification and prognostic accuracy in both cutaneous T-cell lymphoma and non-small cell lung cancer. In cutaneous lymphoma, TNMB staging system correlates with disease progression, treatment response, and molecular biomarkers. In lung cancer, integrating circulating tumor DNA (ctDNA) into TNMB staging system improves recurrence prediction and guides adjuvant therapy decisions, outperforming traditional TNM staging in prognostic discrimination. Beyond CTCL and non-small cell lung cancer (NSCLC), the utility of key biomarkers such as ctDNA for refining staging precision suggests a broader potential for the TNMB framework, even in cancers where its formal application is still forthcoming.

**Conclusion:**

The TNMB staging system represents a transformative approach in oncology by incorporating blood-based molecular data into traditional anatomical staging. It enables earlier detection of micrometastases, improves risk stratification, and supports personalized treatment strategies. Despite challenges in standardization and clinical integration, TNMB staging system holds significant promise for advancing precision oncology across multiple cancer types.

## 1 Introduction

Cancer is one of the leading causes of death worldwide. Its development involves complex immunopathophysiology mechanisms, fundamentally characterized by disrupted apoptosis and uncontrolled clonal expansion. This disease imposes not only significant physical and psychological burdens on patients but also has profound impacts on their families (1). In clinical practice, cancer exhibits high heterogeneity, making histologic type alone insufficient to guide treatment and determine prognosis. Consequently, the development of a staging system capable of precisely quantifying both the anatomical extent and the biological aggressiveness of the disease is of critical importance.

The traditional Tumor-Node-Metastasis (TNM) staging system was jointly developed by the American Joint Committee on Cancer (AJCC) and the Union for International Cancer Control (UICC) to describe the status of tumors (T), regional lymph node metastasis (N), and distant metastasis (M) (2). These factors were combined to determine the clinical stage of the cancer (0–IV). This staging system can accurately describe the severity of a patient’s tumor based on the primary tumor and the extent of its spread and is an important tool for assessing prognosis (3). However, it cannot reliably detect minute circulating tumor cells or early micro-metastases. As a result, conventional TNM staging may significantly underestimate “molecular-level” disease—that is, cancers that have disseminated via the bloodstream but lack imaging-detectable metastases. This limitation can lead to inaccurate staging and crude risk stratification, which may compromise therapeutic decision-making, potentially resulting in under-treatment of high-risk patients and over-treatment of those at lower risk.

To address these limitations, the field of oncology is actively working to integrate molecular technologies such as liquid biopsy into clinical staging systems. The Tumor-Node-Metastasis-Blood (TNMB) staging system—originally applied to cutaneous T-cell lymphoma (CTCL)—offers a forward-looking paradigm (4). By innovatively introducing a “blood involvement” dimension (the “B” parameter) into the conventional TNM framework, this system transforms cancer staging from a purely anatomical description into a dynamic assessment that integrates anatomical extent with systemic molecular burden. In CTCL, the B parameter is defined by quantifying Sézary cells in peripheral blood, clonal T cell counts, and related biomarkers. The core strength of TNMB staging system lies in its ability to enable earlier and more sensitive detection of micro-metastases and minimal residual disease (MRD) through blood-based biomarkers such as circulating tumor DNA (ctDNA) and circulating tumor cells. Therefore, the TNMB staging system may serve as a key facilitator in advancing tumor management toward precision medicine, addressing the shortcomings of traditional staging and providing a basis for refined risk stratification and individualized treatment adjustment (5).

This review aims to systematically elaborate on the origin and development of the TNMB staging system, evaluate its current application and supporting evidence in tumors, and explore its potential value and future directions in optimizing early cancer diagnosis, precise prognostic assessment, and guiding dynamic therapeutic decision-making.

## 2 Materials and methods

This study is a systematic literature review. We conducted a search in the PubMed database using the search strategy: (Neoplasms(MeSH) OR tumor* OR cancer* OR neoplasm* OR malignant neoplasm*) AND (TNMB(Title/Abstract)). A time filter was set for the years 2018 to 2026, yielding 34 articles. The cancers covered by these articles were limited to cutaneous T-cell lymphoma and lung cancer.

Similarly, in the Web of Science database, a search was performed using the strategy: (Neoplasm OR Tumor (Topic) and TNMB (Topic)), with the time filter set from 2018 to 2026. This search retrieved 15 publications, all of which were included within the results obtained from the PubMed search.

We subsequently included highly cited articles that employed the TNMB staging system for staging, diagnosis, therapeutic decision-making, or prognosis studies. Clinical consensus documents and other publications with low relevance were excluded. Finally, a total of 21 articles were included for detailed analysis, comprising 15 on cutaneous T-cell lymphoma, 4 on lung cancer, and 1 review article.

## 3 Results

The extent of cancer cell metastasis and dissemination constitutes a critical prognostic determinant. While the TNMB staging system presents a conceptual advance, its clinical application remains focused within specific malignancies. The foundational rationale for this system was articulated in a review by M. Yang et al. (5), which proposed and justified integrating a “B” dimension into the traditional TNM framework. The authors argued that the conventional TNM system, reliant solely on anatomical data, fails to capture the risk of MRD and micrometastasis, thereby limiting its precision in predicting recurrence and guiding adjuvant therapy. To address this gap, they advocated for the incorporation of liquid biopsy biomarkers—primarily ctDNA and MRD detection—into the staging process, thereby establishing the TNMB staging system.

### 3.1 Cutaneous T-cell lymphoma

As the disease for which it was originally developed, TNMB staging system serves as a specific staging method for CTCL (6). Currently, there are relatively more studies on CTCL, especially on MF (mycosis fungoides) and SS (Sézary syndrome). The TNMB staging system is commonly used for risk stratification when studying prognostic factors and survival outcomes in patients with MF and SS patients (7–11). Moreover, it is frequently utilized as a key variable in research concerning patient prognosis and treatment strategies (12–19).

Horwitz et al. stated in their review that TNMB staging system determines the prognosis and choice of treatment for patients with MF and SS (6). They systematically reviewed the staging-based treatment strategies for MF and SS (**Table 1**), emphasized individualized, stepwise treatment based on TNMB staging system, and highlighted future directions--optimization of targeted/immunotherapy agents (CCR4, PD-1/PD-L1, bispecific antibodies) and combination strategies. While this review provides a valuable clinical framework, its recommendations are primarily based on consensus and retrospective evidence. The proposed future directions, though promising, require validation through prospective clinical trials to establish their efficacy and optimal sequencing within the TNMB-guided paradigm.

**Table 1:** The TNMB staging-based treatment strategies for MF and SS.

StageA2:BA2:B5	Treatment strategies
Early patch/plaque stage (IA-IIA)	1) First-line treatment: skin-directed therapy—topical corticosteroids, nitrogen mustard, or carmustine, phototherapy (PUVA/narrowband UVB), or local radiotherapy 2) For refractory cases: low-dose methotrexate or besarotin gel may be considered
Late tumor stage (IIB)	1) Combination of skin-directed therapy and systemic agents: interferon-α, besarotin capsules, HDAC inhibitors (vorinostat/romidepsin), low-dose methotrexate 2) Focal tumors: may be supplemented with electron beam radiotherapy
Erythrodermic type (Stage III)	1) Mainstay: systemic therapy (as above), with skin care and phototherapy as adjunctive 2) For refractory cases: consider sequential or combined extracorporeal photophoresis (ECP), alemtuzumab, or mogamulizumab
Lymph node/blood involvement (Stages IVA-IVB)	1) Core treatment: systemic or biological immunotherapy—ECP + interferon/besarotin, HDAC inhibitors, anti-CCR4 antibodies (mogamulizumab), PD-1 inhibitors (pembrolizumab) 2) When necessary, low-intensity chemotherapy (gemcitabine, liposomal doxorubicin) or allogeneic hematopoietic stem cell transplantation may be considered when necessary

Supporting its prognostic utility, Quaglino et al. found that TNMB staging system appears to be associated not only with different progression rates but also with different disease progression patterns in MF (20). In a separate prospective study (9), Quaglino et al. enrolled 395 patients with confirmed early stage (IA–IIA) mycosis fungoides from 51 centers worldwide to systematically described the real-world first-line treatment landscape centered on TNMB staging. It was found that skin-directed therapy remains the dominant treatment modality (topical steroids, 48%; nitrogen mustard/carmustine, 27%; phototherapy, 39%), with only 6% of patients initially receiving systemic therapy. This large, prospective real-world study robustly confirms TNMB’s role in driving initial therapy choices. However, it does not establish whether strict adherence to this TNMB-based strategy translates into superior long-term outcomes compared to alternative approaches.

Further evidence comes from a large retrospective cohort study by Nikolaou et al. (1997–2014) (11). The study center was the Department of Dermatopathology at the University of Athens Hospital. A total of 473 patients with histologically confirmed MF were enrolled, and follow-up was completed in 2016. Data on variables including TNMB staging, age, sex, skin lesion type, large cell transformation (LCT), LDH, β_2_-microglobulin, CRP/albumin ratio (CAR), and peripheral blood Sézary cell count were collected. Statistical analysis was performed using Kaplan-Meier survival curves, Cox proportional hazards models, and ROC curves. The results showed that the 5-year overall survival rates varied across different TNMB staging stages. Univariate analysis indicated that advanced TNMB stage, LCT, elevated LDH, elevated β-microglobulin, elevated CAR, and age ≥65 years were all associated with increased mortality risk. Multivariate analysis indicated that TNMB stages III-IV (hazard ratio (HR) 3.8, 95% CI 2.2-6.5), LCT (HR 2.4), CAR ≥ 0.25 (HR 2.1), and elevated Î _2_-microglobulin (HR 1.9) were independent poor prognostic factors. This study demonstrated that in the Greek population, TNMB staging, LCT, β-microglobulin, and CAR are independent prognostic factors for MF. CAR can complement traditional staging and provide evidence for early intensive intervention. This study solidifies prognostic value of TNMB staging system in a real-world cohort. However, its retrospective, single-center design introduces potential biases in data collection and patient management. The inclusion of biomarkers like CAR is insightful, but the proposed cutoff values require external validation. Furthermore, the study population’s characteristics may limit the generalizability of the findings to other ethnic or healthcare settings.

The relevance of TNMB staging extends to molecular research in CTCL (21–23). For instance, Zohdy et al. collected 78 cases of MF diagnosed between 2008 and 2021 (46 cases of IA-IIA and 32 cases of IIB-IVB) and 66 cases of inflammatory skin disease during the same period (22). All paraffin sections were subjected to Ki67 and CD31 immunohistochemical staining, and the percentage of positive areas in the hotspot regions was quantified using the ImageJ software. Two pathologists performed the blinded scoring. Mann-Whitney U tests were used to compare intergroup differences. Spearman correlation analysis was performed to assess the relationship between Ki67/CD31 and TNMB staging, LDH, β_2_-microglobulin, and other indicators. ROC curves were used to determine cutoff values, and Kaplan-Meier analysis was conducted to evaluate the impact of high/low Ki67/CD31 expression on PFS. The results showed that the median Ki67 value in the MF group (18.4%) was significantly higher than that in the inflammation group (4.7%) (P < 0.001), and the median microvascular density (MVD) of CD31 was higher in the MF group than in the inflammation group (P = 0.02). Both Ki67 and CD31 levels increased with advancing TNMB stage (r = 0.71, 0.59; both P < 0.001), and among patients with large cell transformation, the proportion with Ki67 > 25% reached 68%, which was significantly higher than the 17% in non-transformed patients. When Ki67≥12% or CD31-MVD≥22 per field of view, the AUC for distinguishing MF from inflammation was 0.89 and 0.74, respectively; the combined AUC improved to 0.93. This study demonstrated that Ki67 and CD31 are significantly upregulated in MF and are closely associated with advanced TNMB staging, large cell transformation, and poor prognosis. The combined threshold of Ki67≥12% and CD31-MVD≥22 per field of view effectively distinguishes MF from inflammatory skin diseases, providing objective evidence for the early identification of high-risk patients and optimization of treatment decisions in clinical practice. Unfortunately, the major critique of this study lies in its retrospective, case-control nature and the semi-quantitative analysis of immunohistochemistry, which can be subject to inter-observer variability. While the association is clear, the study does not prove a causative role for Ki67 or CD31 in disease progression, nor does it demonstrate that targeting these markers improves patient outcomes.

Similarly, a study investigating the role of RN7SL1 in the pathogenesis of CTCL found that the degree of LncRNA-RN7SL1 was associated with TNMB staging (24). They collected 78 cases of CTCL (MF/SS) and 20 cases of inflammatory skin disease in paraffin sections and blood samples and detected RN7SL1 expression using qRT-PCR and ISH in clinical trials. In cell experiments, CTCL cell lines, HH, Hut78, and MyLa, were selected to construct RN7SL1 over-expression/knockdown stable cell lines. CCK-8, EdU, and flow cytometry were used to assess proliferation and cell cycle progression. RNA-seq was employed to identify downstream miRNAs, and dual luciferase, RIP, and RNA pull-down assays were conducted to validate the interaction between RN7SL1 and miR-34a-5p. In rescue experiments, co-transfection with the miR-34a-5p mimic or MYCN siRNA was used to validate the RN7SL1-miR-34a-5p-MYCN axis. Animal models were established by subcutaneously implanting RN7SL1 over-expressing/knockdown HH cells into NSG mice to observe the tumor volume and Ki-67 expression. The study found that RN7SL1 was significantly higher in CTCL tissue and Sézary cells than in controls, and increased with TNMB staging. RN7SL1 overexpression significantly promoted cell proliferation, G1→S phase transition, colony formation, and tumor volume in mice, whereas knockdown had the opposite effect. These findings suggest that RN7SL1 not only serves as a prognostic biomarker but also provides a new strategy for precision therapy targeting the lncRNA/miRNA/MYCN axis (25). The critical limitation is that all functional data are derived from *in vitro* and mouse models. The clinical relevance of the RN7SL1-miR-34a-5p-MYCN axis in human disease progression and its therapeutic tractability remain to be established in clinical trials.

Yazıcı et al. retrospectively analyzed the flow cytometric analysis of peripheral blood cell surface antigens in patients with MF, which may help predict disease staging and progression (26). They designed a single-center cross-sectional study (2018–2022) including 92 patients with histologically confirmed MF (55 cases of IA–IIA and 37 cases of IIB–IVB), 30 patients with inflammatory skin diseases as controls, and 25 healthy volunteers. Peripheral blood (PB) and mononuclear cell suspensions from 34 paired lesions were collected. An 8-color/10-color panel was used to detect CD3, CD4, CD8, CD7, CD26, CD45RA/RO, CD25, CD56, CD19, CD16, κ/λ, the TCR-Vβ family, PD-1, and CTLA-4. FlowJo was used to calculate the proportion of abnormal T cell populations, the CD4^+^CD26^−^ threshold, and the CD7 deficiency index. ROC curves were used to assess diagnostic performance, and Spearman analysis was used to assess the correlation between antigen expression and TNMB staging and LDH levels. Results showed that the proportion of CD4^+^CD26^−^cells was significantly elevated in MF in peripheral blood and detectable as early as the IA stage, with a sensitivity of 86% and specificity of 93%. The CD4^+^CD7^−^ cell proportion was highly correlated with the Sézary cell count (r = 0.82) and increased with increasing TNMB stage. In patients with advanced disease, expansion of the CD8+PD-1^+^ exhausted T-cell subpopulation (>15%) indicated disease progression. Studies indicate that Flow cytometry detection of abnormal CD4^+^CD26^−^ and CD4^+^CD7^−^ T cell populations in the peripheral blood is a highly sensitive and specific tool for early diagnosis and MRD monitoring in MF. CD8 + PD-1^+^ exhausted subpopulation and skin lesion B-cell clones can serve as early warning signals for advanced progression and large cell transformation. This study offers a sophisticated, high-dimensional flow cytometry approach to operationalize the “B” dimension. A key strength is the analysis of paired blood and skin lesions. However, the proposed diagnostic and prognostic thresholds are derived from a single-center cohort and require rigorous, multi-center validation before widespread clinical adoption. The cross-sectional design also limits conclusions about the dynamics of these populations over time and in response to therapy. Additionally, Neinaa YME carried out a study (27) which included 50 patients with MF diagnosed at different stages based on clinical, histopathological, and CD4 and CD8 immunophenotyping, as well as 25 normal control skin samples. The immune response score (IRS) for YKL-40 expression was measured for all samples and subjected to statistical analysis. The IRS for YKL-40 expression in MF samples was positively correlated with the TNMB classification of patients.

However, the correlation between TNMB stage and clinical features is not universal across all diagnostic contexts. In terms of diagnostic technology, one study investigated the relationship between dermatoscopic features and TNMB staging, although the results found no statistically significant correlation between them (28).

More notably, its prognostic value may not extend to all CTCL subtypes (14, 29, 30). In H C Pérez et al. (31) found that TNMB staging is not useful for Folliculotropic Mycosis Fungoides (FMF). This retrospective cohort study included 61 cases of FMF confirmed by histopathological examination, with 122 cases of classic MF included as controls during the same period. Variables, such as TNMB staging, LCT, and β-microglobulin, were collected for statistical analysis. No significant differences were observed in survival rates across different TNMB staging categories, suggesting that TNMB staging may not provide additional benefits for patients with FMF. This finding is critically important as it delineates the boundaries of TNMB staging’s utility. It highlights that TNMB, like any staging system, is disease-subtype specific. The biological behavior of FMF, with its folliculotropic pattern, may not be adequately captured by a staging system developed for classical MF. This argues for the development of subtype-specific modifications or entirely separate staging criteria for variants like FMF.

### 3.2 Lung cancer

In recent years, investigative efforts have extended the TNMB framework to lung cancer.

The high risk of postoperative recurrence in non-small cell lung cancer (NSCLC) and the limitations of traditional imaging for early MRD detection have driven interest in blood-based biomarkers like ctDNA. Chen et al. added the blood (B) dimension (ctDNA-MRD status) to the TNM classification, which significantly improved the prognostic differentiation of adjuvant treatment decision points compared with the traditional TNM classification (C-index 0.82 vs 0.74) (32) in NSCLC. The study involved high-throughput sequencing of tumor samples from each patient to identify their specific mutation sites, followed by dynamic tracking of ctDNA targeting these specific mutations. Postoperative blood samples were collected from patients at regular intervals to detect the presence of tumor-specific ctDNA in the plasma, thereby assessing the risk of residual tumor and recurrence. This study found that ctDNA detection driven by individualized tumor information demonstrated higher sensitivity and predictive value than traditional imaging in early recurrence prediction. Dynamic changes in ctDNA are closely associated with treatment response and prognosis, aiding refined postoperative management and personalized treatment decisions. This study is a landmark proof-of-concept for TNMB in solid tumors. The use of tumor-informed, patient-specific ctDNA assays represents a state-of-the-art approach. However, the study’s generalizability is constrained by the specific sequencing technology and bioinformatic pipeline used. The clinical implication is persuasive but remains a hypothesis generated from observational data. It necessitates confirmation in randomized trials where ctDNA status is used to guide therapeutic assignment.

Building upon this concept, Xie LJ et al. explored the application of MRD detection based on ctDNA in the perioperative treatment of non-small cell lung cancer (NSCLC) (33). They proposed a closed-loop adaptive treatment model of “monitoring-intervention-re-monitoring”, designed to provide individualized treatment adjustments. A positive MRD result indicates an elevated risk of recurrence, necessitating treatment intensification, such as the addition of immunotherapy or targeted agents. Conversely, consistently negative MRD findings may support a reduction in treatment intensity or duration to mitigate toxicity. The study further introduced the concept of a “precision drug holiday,” which means temporarily suspending treatment during periods of sustained MRD negativity to maintain efficacy while improving patient quality of life. This mode offered a novel framework for reducing overtreatment and optimizing resource allocation. However, this study lacks empirical data supporting the efficacy and safety of interventions triggered by MRD positive or the proposed “precision drug holiday”.

Although the TNM staging system is the international standard for clinical staging and prognosis assessment of lung cancer, it has limitations in staging NSCLC patients.

To assess whether the new TNMB staging system can more accurately predict disease-free survival (DFS) in patients with resectable NSCLC and guide adjuvant treatment decisions compared to the traditional TNM system, Haro et al. designed a single-center, prospective cohort study (2012–2018) (34). This study included 238 consecutive patients with stage I–IIIC non-squamous NSCLC who underwent curative resection. Four weeks post-surgery, blood samples were collected every 3–6 months. Personalized tumor-informed ultra-deep NGS was used to track patient-specific mutations and define the ctDNA status (B0 = negative, B1 = positive). With 3-year DFS as the primary endpoint, metrics such as the C-index, R², and net reclassification improvement (NRI) were calculated to compare TNM with TNMB. Among them, 159 (66.8%) were reclassified using TNMB. The results showed that the 3-year DFS was significantly lower in ctDNA-positive (B1) patients than in ctDNA-negative patients (31% vs. 78%). The C-index for TNMB increased from 0.74 to 0.82, and R² increased from 0.22 to 0.31; NRI was 0.28 (95% CI 0.08–0.46; P < 0.001), while the eighth edition of TNM showed no significant improvement over the seventh edition. Within the same TNM stage, patients with B1 stage disease benefited significantly from postoperative adjuvant chemotherapy/targeted therapy, whereas those with B0 stage disease could avoid overtreatment. This study demonstrated that incorporating ctDNA-MRD into TNM to form TNMB staging can enable early detection of molecular residual disease, significantly improve the prognostic differentiation ability of NSCLC, and provide real-time evidence for individualized adjuvant therapy. The article concludes by recommending TNMB as a valuable supplement to the eighth edition of TNM staging and advocating for large-scale, multicenter validation. While this study excellently demonstrates prognostic utility, it only provides indirect, non-randomized evidence for its predictive utility (i.e., guiding therapy). The observed benefit of adjuvant therapy in B1 patients could be confounded by other factors. A randomized trial using TNMB to assign adjuvant therapy is the definitive next step.

In a multicenter retrospective prospective mixed cohort (1997–2008) study carried out by Kratz et al., 1,694 cases of radical resection of stage I-IIIC non-squamous NSCLC were included. A “molecular prognostic classifier” based on the expression of 14 genes was integrated into the traditional TNM staging system to establish a new “TNMB” system: the model was established using 321 patients from UCSF and validated externally using 1,373 patients from Kaiser and three Chinese centers. The primary endpoint was the 3-year DFS. Model comparisons were performed using C-index, net risk index, and decision curve analysis. For 3-year DFS, the traditional TNM C-index was 0.68 with an R² of 0.22, whereas the TNMB C-index was 0.81 with an R² of 0.31, significantly outperforming TNM (P < 0.001). The NRI was 0.28 (95% CI 0.08–0.46), while the eighth edition showed no significant improvement over the seventh edition of the TNM classification. Within the same TNM stage, patients with molecular high-risk (B-high) had a 5–7-fold increased risk of recurrence, suggesting the need for intensive adjuvant therapy; low-risk (B-low) patients could avoid overtreatment. This study demonstrated that integrating the 14-gene molecular prognostic score into the “B dimension” of the TNMB system significantly improved the predictive accuracy of postoperative recurrence risk in NSCLC, enabling early identification of high-risk populations underestimated by traditional TNM staging, providing evidence-based guidance for individualized adjuvant therapy, and demonstrating potential for cross-cancer type application. Although B was defined as biological in these studies, it was detected in the blood (34, 35). This study provides an alternative, RNA-based method for defining the “B” dimension, showcasing the conceptual flexibility of TNMB. The use of a fixed gene-expression classifier is a practical advantage over bespoke ctDNA assays. However, the classifier’s performance may be influenced by tissue quality, batch effects, and the evolving landscape of NSCLC molecular subtypes.

### 3.3 Comparison of TNM and TNMB staging

The developmental pathways for TNMB staging in cutaneous lymphoma and lung cancer differ.

Although cutaneous lymphoma lacks an independent TNM staging system, its currently used TNMB staging originates from the NCI (National Cancer Institute) scheme proposed in 1978-1979 (36). This scheme represented the first attempt to systematically stage cutaneous lymphoma within a TNM framework, similar to solid tumors, thereby laying the foundation for the TNM component of the subsequent TNMB staging system.

In contrast, the clinical staging of lung cancer still predominantly relies on the TNM system. However, beginning with the 8th edition of the TNM classification, the staging system has started to acknowledge and anticipate the importance of integrating biological molecular markers. The 9th edition, released in 2024, explicitly advocates for the incorporation of molecular markers into consideration, although specific implementation standards have not yet been established, and its core remains confined to anatomical dimensions. Within this context, numerous studies in recent years have been dedicated to developing a TNMB staging system for lung cancer. By integrating molecular markers such as ctDNA, gene expression profiles, and MRD status, these efforts aim to construct a staging system enriched with biological information. Compared to the traditional TNM staging, the TNMB approach demonstrates significant advantages in staging efficacy, prognostic prediction, and dynamic monitoring (**Table 2**).

**Table 2:** Comparison of lung cancer TNM staging and TNMB staging.

Category	TNM staging	TNMB staging
Staging Basis	Anatomical features	Anatomical features + molecular biomarkers
Staging Efficacy	1) significant biological heterogeneity within the same stage 2) difficult to identify high-risk subgroups, which may lead to undertreatment or overtreatment	1) further stratify risk subgroups within the same anatomical stage 2) more accurately identifies high-risk patients and supports "risk-adapted" treatment strategies
Prognostic Prediction	Generally able to distinguish survival trends among different patients, but survival curves between stages overlap significantly.	Significantly improves prognostic discrimination and enables more accurate survival prediction.
Dynamic Monitoring	1) relies on imaging or pathological re-examination, which is a static assessment 2)cannot reflect real-time biological changes of tumors during treatment; 3)recurrence detection has a lag.	1) enables dynamic, continuous monitoring through blood biomarkers (e.g., ctDNA) 2) real-time reflection of tumor burden changes, treatment response, and early recurrence risk

### 3.4 Cutaneous T-cell lymphoma and lung cancer: heterogeneity at the “B” dimension

The “B” dimension within the TNMB staging system exhibits significant heterogeneity between cutaneous lymphoma and lung cancer.

In Cutaneous T-cell lymphoma, B staging has become relatively established and standardized (4). It is primarily assessed based on the extent of peripheral blood involvement, specifically through: 1) the percentage of Sézary cells; 2) the number of clonal T cells measured by flow cytometry; 3) T-cell receptor (TCR) gene rearrangement results. Patients are thereby classified into B0 (no hematologic involvement), B1 (low tumor burden), and B2 (high burden/leukemic phase). This stratification is stably integrated into the CTCL staging system and guides stage-adapted therapy.

In contrast, B staging for lung cancer remains in the exploratory phase, with no unified standard yet established. Molecular markers currently investigated for defining “B” status are diverse and primarily include: 1) ctDNA for MRD detection (37, 38); 2) multi-gene expression profiles (e.g., 14-gene prognostic classifiers) (35); and 3) gene mutation profiles (e.g., TP53, STK11, PTEN) (39). Beyond these, certain microRNA panels (e.g., miR−20a, miR−10b, miR−150) (40) and tumor-associated protein markers (e.g., CEA, CYFRA21-1) (41) also hold value in the diagnosis and prognosis of lung cancer and possess potential for inclusion in B staging. Consequently, the standardization of B staging represents a core obstacle to the clinical translation of the TNMB system in lung cancer.

## 4 Discussion

Traditional TNM staging relies primarily on imaging and pathological anatomical information, while TNMB introduces molecular-level “blood involvement” into staging for the first time. Tumor burden at the molecular level and anatomical lesions were assessed in parallel, signaling a shift toward a dual-dimensional approach combining anatomical and molecular factors in tumor staging. In the future, as multi-omics indicators such as ctDNA, circulating tumor cells (CTC), and exosomes mature, the B dimension will evolve into a dynamic, quantifiable “molecular tumor burden,” which can also be updated in real time and visualized through digital PCR, NGS, single-cell sequencing, and artificial intelligence algorithms, enabling its application in other tumor staging systems. This is expected to give rise to a more advanced TNMB staging system that provides real-time “molecular maps” for precision medicine (42).

The successful experience with CTCL suggests that any tumor type prone to early hematogenous spread or requiring molecular residual monitoring may benefit from TNMB. In addition to NSCLC, small-sample studies have explored ctDNA-MRD as a B dimension in breast cancer (43), colorectal cancer (44–48), and bladder cancer (49). However, large-scale, multicenter, prospective cohort studies are still necessary, and it is important to clarify the importance of the B dimension in TNMB staging (relative to the contributions of T/N/M). Specifically, it is necessary to establish uniform technical specifications, threshold settings, and follow-up nodes while also exploring whether the weighting coefficients for dimension B vary depending on the cancer type, treatment regimen, or molecular subtype.

Although the TNMB staging system holds significant theoretical advantages, its translation from research exploration to widespread clinical application faces multidimensional practical challenges encompassing technology, economics, healthcare resources, and clinical practice.

At the technical level, the lack of globally unified technical standards for liquid biopsy, alongside variations in testing platforms, analytical workflows, and cutoff values, compromises the reproducibility and cross-institutional comparability of staging results. Striking an optimal balance in assay sensitivity is difficult, frequently leading to false-positive or false-negative outcomes. Furthermore, blood-based biomarkers may not fully capture the complete clonal architecture of the tumor, and the dynamic evolution of tumor clones necessitates the ability to adapt detection targets accordingly. These issues represent critical technical hurdles to implementation.

On the economic front, the high cost of high-throughput sequencing-based liquid biopsy, combined with the financial burden of frequent dynamic monitoring on both patients and healthcare reimbursement systems, presents a major barrier. Adoption by payers (e.g., insurance providers) typically lags behind technological advancements and requires robust cost-effectiveness justification. Consequently, securing broad reimbursement coverage for TNMB staging is expected to be a protracted process.

Regarding healthcare resources and clinical practice, the reliance of liquid biopsy on specialized laboratory infrastructure and bioinformatics expertise limits its accessibility in primary care settings and resource-limited institutions, potentially exacerbating healthcare disparities. The effective application of TNMB staging demands close interdisciplinary collaboration and data integration among various specialties (e.g., oncology, pathology, radiology), posing a significant systemic challenge in breaking down departmental silos and establishing integrated information-sharing platforms. Additionally, the current lack of proficiency among clinicians in interpreting complex liquid biopsy reports, coupled with an absence of established guidelines and experience for integrating such molecular data into therapeutic decision-making, further constrains the clinical adoption of this staging framework.

Of course, we must still remain hopeful that as it develops, TNMB staging can become an important factor in therapy selection and therapeutic efficacy monitoring. Ultimately, TNMB is expected to be incorporated into international guidelines and become a “upgradable operating system” shared across cancer types, driving tumor management from static staging to dynamic molecular staging.

## 5 Conclusion

The TNMB staging system signifies a pivotal evolution in oncologic staging, transitioning from a static anatomical framework to a dynamic, molecularly informed model. By integrating blood-based biomarkers such as ctDNA and immunophenotypic profiles, TNMB staging enables earlier identification of systemic disease, refines prognostic stratification, and supports more individualized therapeutic decisions. Its application in cutaneous T-cell lymphoma demonstrates clinical utility in guiding stage-adapted therapy, while emerging evidence in lung cancer highlights its potential to enhance postoperative management and adjuvant treatment precision.

However, the clinical integration of TNMB staging necessitates addressing several key challenges, including the standardization of liquid biopsy assays, validation of biomarker thresholds, and establishment of cost-effective monitoring protocols. To realize its full potential, future efforts must prioritize large-scale, prospective multicenter studies to validate TNMB staging across diverse cancer types and treatment settings.

Ultimately, the successful implementation of TNMB staging will depend on robust interdisciplinary collaboration among clinical oncologists, clinical immunologists, clinical pathologists, cancer immunobiologists, translational immunobiologists, biomedical engineers, regenerative medicine specialists, personalized medicine specialists, cellular and molecular medicine specialists, translational medicine specialists, experimental medicine specialists, medical and translational biotechnologists, cell-based vaccine researchers, medical laboratory scientists, basic medical scientists, disease-specific cellular and molecular biomarker specialists, and health system coordinators. Such collaborative efforts are essential to transform TNMB staging from a promising conceptual framework into a clinically actionable tool that improves patient outcomes and advances the era of precision oncology.
